# Structure-based discovery of selective CYP_17_A_1_ inhibitors for Castration-resistant prostate cancer treatment

**DOI:** 10.1093/biomethods/bpab026

**Published:** 2021-12-25

**Authors:** Damilola A Omoboyowa, Toheeb A Balogun, Oluwatosin A Saibu, Onyeka S Chukwudozie, Abdullahi Alausa, Samuel O Olubode, Abdullahi T Aborode, Gaber E Batiha, Damilola S Bodun, Sekinat O Musa

**Affiliations:** 1 Department of Biochemistry, Adekunle Ajasin University, Akungba-Akoko, Nigeria; 2 Department of Environmental Toxicology, University of Duisburg-Essen, North Rhine-Westphalia, Germany; 3 Division of Biological Science, University of California San Diego, CA 92161, USA; 4 Department of Biochemistry, Ladoke Akintola University of Technology, Ogbomoso, Oyo State, Nigeria; 5 Healthy Africans Platform, Research and Development, Ibadan, Nigeria; 6 Department of Pharmacology and Toxicology, Faculty of Veterinary Medicine, Damanhour University, Damanhour City, Egypt

**Keywords:** prostate cancer, CYP_17_A_1_, almond, androgen, molecular docking

## Abstract

Prostate cancer (PCa) is the most common malignancy found in men and the second leading cause of cancer-related death worldwide. Castration-resistant PCa (CRPC) is defined by PCa cells that stop responding to hormone therapy. Cytochrome P450 17α-hydroxylase/17,20-lyase (CYP17A1) plays a critical role in the biosynthesis of androgens in humans. Androgen signaling cascade is a principal survival pathway for PCa cells and androgen-deprivation therapy (ADT) remains the key treatment for patients marked with locally advanced and metastatic PCa cells. Available synthetic drugs have been reported for toxicity, drug resistance, and decreasing efficacy. Thus, the design of novel selective inhibitors of CYP17A1 lyase would help circumvent associated side effects and improve pharmacological activities. Therefore, we employed structural bioinformatics techniques via molecular docking; molecular mechanics generalized born surface area (MM-GBSA), molecular dynamics (MD) simulation, and pharmacokinetic study to identify putative CYP17A1 lyase inhibitors. The results of the computational investigation showed that the *Prunus dulcis* compounds exhibited higher binding energy than the clinically approved abiraterone acetate. The stability of the ligand with the highest binding affinity (quercetin-3-o-rutinoside) was observed during MD simulation for 10 ns. Quercetin-3-o-rutinoside was observed to be stable within the active site of CYP17A1Lyase throughout the simulation period. The result of the pharmacokinetic study revealed that these compounds are promising therapeutic agents. Collectively, this study proposed that bioactive compounds from *P. dulcis* may be potential selective inhibitors of CYP17A1Lyase in CRPC treatments.

## Introduction

Prostate cancer (PCa) is a global challenge and the second leading cause of cancer-related deaths in men and the fifth most common cause of death worldwide [1]. Medical reports have projected an exponential increase of PCa cases diagnosed with a survival rate of 3% in advanced metastatic PCa disease. Huggins [2] reported that PCa cell proliferation depends critically on the presence of androgens. The first generation treatment method for PCa cells in affected men is androgen-deprivation therapy (ADT) [3]. The use of luteinizing releasing hormone such as Lupron for chemical-mediated castration has also proven to be an excellent therapeutic intervention [4]. Disappointedly, this therapy is only effective for a limited period by reducing the testosterone level by 90% of production in the testes and does not have any overlapping effect on the remaining 10% testosterone produced in the adrenal gland [5]. After approximately one and half years of treatment, androgen receptors will start to be stimulated and respond to the low level of testosterone and other androgens, including progesterone, thereby activating testosterone biosynthesis in large quantities, which ultimately leads to the re-development of PCa growth. The re-emergence of PCa results in metastatic castration-resistant PCa (CRPC) [6]. Due to the severe side effects of CRPC, several new treatment methods to curb this condition are gaining intense consideration.

Previous studies have reported the failure of existing treatment methods such as surgical or medical castration in the treatment of CRPC, thereby driving the need for alternative therapy. Furthermore, the overall survival rate of PCa patients has significantly improved using docetaxel, a second-line chemotherapeutic agent [6]. Ketoconazole is an antibiotic that can alleviate clinical symptoms of PCa via a non-selective inhibition of cytochrome P450 [6, 7]. The cytotoxicity, hepatotoxicity, and genotoxicity associated with each treatment method discourage their long-term usage.

**Figure 1:**
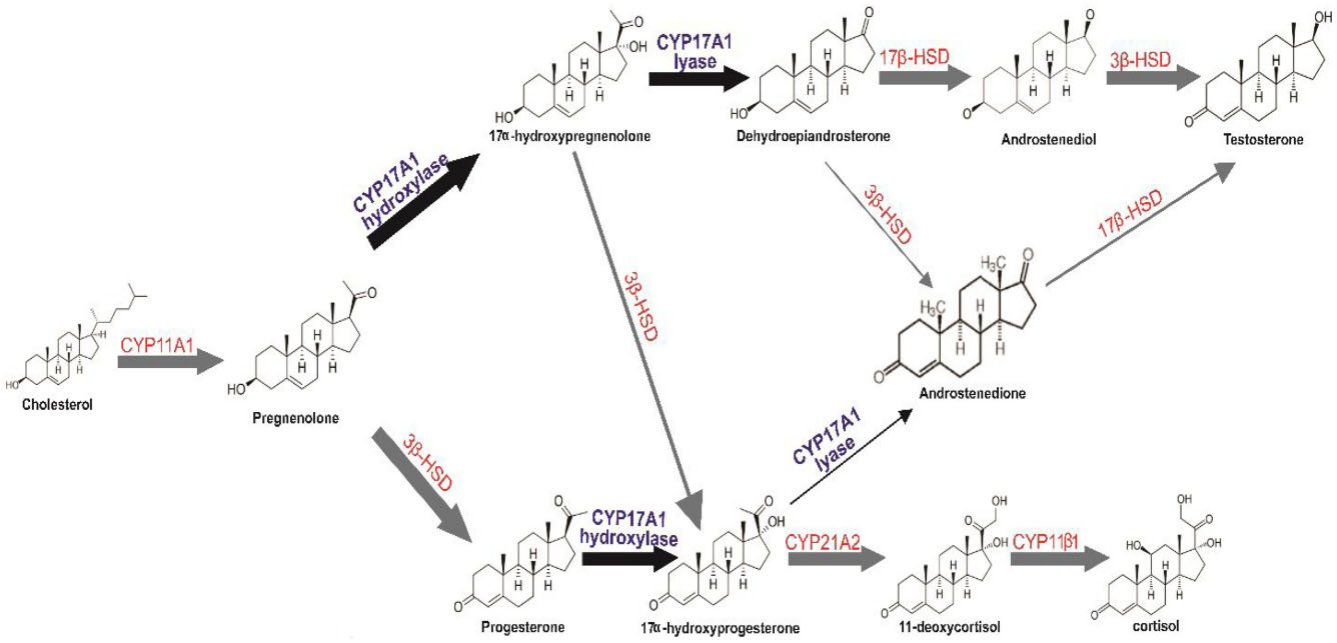
the role of CYP_17_A_1_ in the steroid biosynthesis pathway; the reactions catalyzed by CYP_17_A_1_ are shown in bold and black arrows. HSD, hydroxysteroid dehydrogenase. CYP_17_A_1_ is a bifunctional enzyme that hydroxylates pregnenolone and progesterone in 17α positions of the steroid to produce 17α-hydroxypregnenolone and 17α-hydroxyprogesterone, respectively. CYP_17_A_1_ then employed its C_17,20_-lyase (lyase) functionality to cleave 17α-hydroxypregnenolone to dehydroepiandrosterone (DHEA) and 17α-hydroxyprogesterone to androstenedione. DHEA and androstenedione yield testosterone. Furthermore, testosterone can be converted into potent androgen dihydrotestosterone (DHT) in the prostate. There is a notable production of androgen in prostate tumors of PCa patients [13, 19]. Therefore, CYP_17_A_1_ lyase represents the rate-limiting step in androgen biosynthesis and a critical therapeutic target in CRPC treatment.

The approval of abiraterone acetate (AA; Zytiga) in combination with prednisone supplementation denotes a significant innovative and breakthrough research in PCa. Clinical trial results have shown that AA eliminates the remaining 10% testosterone produced by the adrenal gland, which can catalyze the sustenance of CRPC [8]. Administration of AA-prednisone therapy to PCa patients recorded a four-month survival rate compared with the placebo group, ability to carry out normal daily activities, improved quality of life, and skeletal-muscle-related developments [9, 10]. AA is a steroidal scaffold that selectively inhibits both the hydroxylase and lyase catalytic activity of CYP_17_A_1_. This enzyme is found in the endoplasmic reticulum of both the adrenal glands and testes. The AA inhibitory potential against CYP_17_A_1_ can deter the biosynthesis of androgens needed for tumor growth. Unfortunately, AA exhibits an irreversible competition within the binding site of CYP17A1, which leads to the blockage of CYP_17_A_1_ hydroxylase and lyase functionality [11]. However, due to the CYP_17_A_1_ 17α-hydroxylase inhibition by the AA, there is a reduction in the glucocorticoids biosynthesis, which triggers an elevated level of adrenocorticotropic hormone from the anterior pituitary gland, thus can pose serious side effects that can only be suppressed by co-administration of cortisol replacement prednisone [12–14]. The development of a potent, non-steroidal selective inhibitor of the lyase catalytic property of CYP_17_A_1_ (Fig. 1) would help mitigate the need for corticosteroid supplementation, intolerable response to drug events, and side effect profile. The specific goal of such a drug would be dependent upon its selective inhibition of other cytochrome P450 enzymes in the steroidogenic pathway, including CYP_21_A_2_ and CYP_11_B_1_, to hamper glucocorticoid and mineralocorticoid production in excess [15]. For instance, in a phase three clinical trial using TAK-700 (orteronel) for CRPC therapy, there is moderate selective inhibition of CYP_17_A_1_ lyase [16, 17]. Although it was administered in combination with prednisone depicting a need for more selective inhibitors of other CYPs is paramount. Furthermore, previous studies have reported AA as a non-selective inhibitor of CYP_21_A_2_ and CYP_11_B_1_, which further requires the co-administration of prednisone [18]. Overall, CYP_17_A_1_ inhibition demonstrated significant treatment benefits in CRPC patients through down-regulation of androgen signaling cascades, inducing Pca cell growth [19].

Since time immemorial, natural products have been used in folklore medicine to treat different metabolic diseases due to their antioxidant, antiviral, anticancer, and antidiabetic activity [20, 21]. Bioactive compounds have received global interest for their pharmacological benefit in clinical manifestations due to their affordability, accessibility, and minimal side effect compared with other synthetic drugs. Several potent drug candidates with fewer associated side effects have been synthesized from medicinal plants [22]. Furthermore, phytoconstituents from natural products possess anti-proliferative properties against different tumor cells [23, 24]. Therefore, medicinal plants with anti-cancer activity and solid binding interactions can be used as a template for developing selective inhibitors against CYP_17_A_1_lyase.

The Almond *Prunus dulcis* is a famous tree commonly found in the Mediterranean countries and other hot temperate regions [25]. The Almond plant contains essential bioactive compounds such as polyphenols, terpenoids, alkaloids with diverse biological activity, including antioxidant, immune-stimulant properties, and anti-cancer potential [26]. The Almond plant contains abundant unsaturated fatty acids, mainly oleic and linoleic acid. Various health benefits associated with the consumption of Almond have been reported. Almond nuts help lower low-density lipoprotein (LDL) cholesterol and simultaneously increase the level of high-density lipoprotein cholesterol and prevent diabetes-associated disorders like heart disease and type II diabetes [27, 28]. *Invivo* studies have reported that almond oil significantly reduces colon cancer cells’ emergence and exhibited hepatoprotective properties [29]. To the best of our knowledge, this is the first computational study to evaluate the selective inhibition of Almond *P. dulcis* against CYP_17_A_l_yase by combining molecular docking, MM-GBSA calculation, MD simulation, and pharmacokinetics study.

In this present study, *in silico* approach was utilized to ascertain potential drug-like compounds. Forty compounds of Almond *P. dulcis* were identified from kinds of literature. The compounds’ chemical structures were retrieved from the PubChem database and screened using molecular docking. After examining the pose analysis and MM-GBSA calculation, five lead compounds were obtained. The compound with the highest binding energy was validated for its stability using MD simulation. The compounds demonstrated outstanding pharmacokinetic profiles via *in silico* ADMET analysis. Ultimately, we identified novel compounds that may serve as a new treatment method for CRPC.

## Methodology

### Protein preparation

The 3D structure of the protein co-crystallized with abiraterone was retrieved from Protein Data Bank (PDB ID: 3RUK). The protein preparation wizard of the maestro interface was utilized to refine the protein by fixing missing hydrogen atoms, loops, and terminals. The prepared protein was minimized using the OPLS3 force field while all the added hydrogen was purified at pH 7.0. The optimized protein was selected for molecular docking and MD simulation [30].

### Ligand preparation and grid generation

Forty phytochemicals of Almond *P.dulcis* were obtained from published literature on natural compounds. The structures of the ligands were retrieved from the PubChem database and prepared using the Ligprep module of Maestro [31]. All the ligands were optimized finely and docked into the binding pocket of the protein.

Grid generation is crucial for defining the thumb palm pocket (active site) of CYPA17 lyase. The grid box was generated using the receptor grid generation integrated with the maestro interface. The centroid of the coordinate was 27.2 on the *x*-coordinate, −1.6 on the *y*-coordinate, and 32.18 on *z*-coordinate [32].

### Molecular docking

The forty phytochemicals were docked into the target active site of the protein molecule (3RUK) using Glide extra precision (XP) for the calculation of the binding energy and ligand efficiency [31]. The Maestro ligand interaction tool was used to view the interaction, such as hydrogen bond (HB), hydrophobic, and pi–pi stacking interaction of the docked complex. The prepared ligand library and the co-crystal abiraterone ligand were docked into the binding site of the CYPA17lyase using the standard precision (SP) algorithm, applying a scaling factor of 0.8 and partial charge cutoff of 0.15, the conformation of the ligand was set as flexible. The extra precision (XP) method was further employed to re-rank the binding poses of SP results, thereby minimizing false-positive results [33].

The molecular docking protocol was validated to ensure the accuracy and reliability of the docking analysis. The validation process was achieved by removing the co-crystalized ligand from the protein structure, prepared, and docked back into the active site of CYP_17_A_1_lyase using the docking procedure mentioned earlier. The docked complex was analyzed in terms of root mean square deviation (RMSD), which results in an RMSD value of 1.6 Å. Several reports have shown that the normal range for docking protocol validation varies within an RMSD value of 0–2 Å [34].

### Binding free energy calculations using MM-GBSA

The binding free energy of the docked complexes was calculated using the prime MM-GBSA integrated with the Prime Schrodinger suite. The CYP_17_A_1_lyase-ligands complexes were computed using the VSGB solvation system and OPLS3 force field. Additionally, the sampling model was minimized and the rotamer search algorithm was incorporated. The relative free binding energy was calculated based on the following equation highlighted below [35, 36]

(1) $${\Delta G}^{\mathrm{bind}}=\;G^{\mathrm{complex}\;}-(G^{\mathrm{protein}}+\;G^{\mathrm{ligand}})$$

The molecular docking and MM-GBSA raw data were extrapolated and the graphs were plotted using GraphPad Prism (Version 8) [37].

### ADMET screening

The ADMETSAR web server (http://lmmd.ecust.edu.cn/) was used to predict the Absorption, Distribution, Metabolism, and Excretion properties of the hit compounds. ADMETSAR indicates the pharmacological properties of the ligands according to Lipinski’s rule of five [38, 39]. Quercetin-3-o-rutinoside, which has the highest binding energy and the drug-like property, was selected for further analysis via MD simulations.

### MD simulation

MD simulation analysis was carried out using the Desmond program of Schrodinger software [40]. The docked ligand complex with the highest binding energy was selected for the MD simulation study with OPLS 2005 force-field parameters. The docked complex was centered on the orthorhombic box of the predefined TIP3P water system. The box’s volume was minimized and the net charge of the system was neutralized by incorporating 0.15 M NaCl into each system to mimic the physiological state [41]. The temperature and pressure were kept constant at 300 K and 1.01325 bar using the Nose–Hoover thermostat and Martyna–Tobias–Klein barostat methods. Simulation analysis was performed through the NPT ensembles by considering heavy atoms, time intervals, and pressure [42]. Exactly 10 ns of the flexible system was carried out with NPT ensembles and the long-range electrostatic interactions were computed using the Particle–Mesh–Ewald algorithm. The trajectories were recorded at 4.8 ps intervals and the protein–ligand interaction, stability, and behavior were performed using the Desmond simulation interaction diagram in maestro [40].

## Results and discussion

### Molecular docking, MM-GBSA, drug-like properties, and interaction profiling of CYP17A1Lyase–ligand complexes

**Figure 2:**
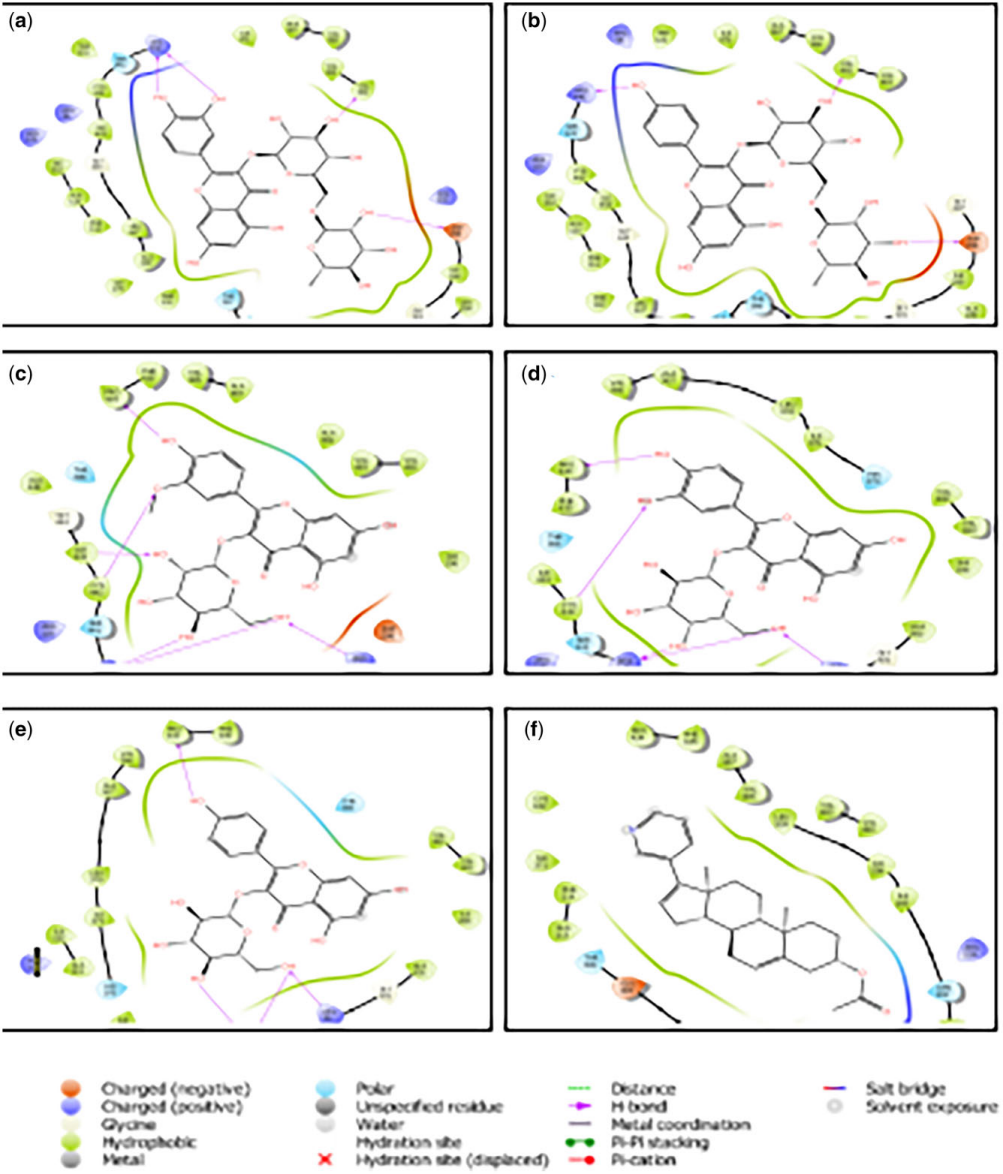
2D molecular contacts profiling of docked compounds with amino acid residues (4.00 Å) at the active site of CYP_17_A_1_ lyase (**a**), quercetin-3-0-rutinoside (**b**), kaempferol-3-o-rutinoside (**c**), isorhamnetin-3-o-galactoside (**d**), quercetin-3-o-galactoside (**e**), and kaempferol-3-o-glucoside (**f**) AA.

After the docking model of 40 compounds derived from Almond *P.dulcis*, the binding affinity (−7.052 kcal/mol) of the standard (AA) was used to set the cut-off value of the screened phytochemicals. Hence, as lower binding energy corresponds to higher binding affinity [43], ligands with lower binding affinity than the referenced compound were filtered out. In contrast, the top five best compounds (quercetin-3-o-rutinoside, kaempferol-3-o-rutinoside, isorhamnetin-3-o-galactoside, quercetin-3-o-galactoside, and kaempferol-3-o-glucoside) based on their higher binding affinity were selected (as shown in Table 1). Figure 2 also depicted the mode of binding of the five complexes and all represented a similar mode of binding compared with the standard. This shows that the binding mechanism with which a co-crystallized ligand (AA) inhibits the target CYP_17_A_1_lyase receptor may also be the exact mechanism responsible for the selected five compounds.

**Table 1:** molecular docking results of bioactive compounds from Almond *P. dulcis* and the reference ligand in terms of binding energy (Kcal/mol), MM-GBSA calculation, the interaction of the compounds with CYP_17_A_1_ lyase, and the drug-like properties

Ligands	Binding energy (Kcal/mol)	MM-GBSA (Δ*G*_BIND_)	ADME properties
Quercetin-3-o-rutinoside	−14.361	−65.24	MW (<500 Da) = 610.52 g/mol Number of HB acceptor (<10) = 16 Number of HB donor (<5) = 10 Log *P*(<5) = 2.43 Violations (ROF) = 3 TPSA = 269.43 Å²
Kaempferol-3-o-rutinoside	−12.645	−58.45	MW (<500 Da) = 594.52 g/mol Number of HB acceptor (<10) = 15 Number of HB donor (<5) = 9 Log *P*(<5) = 0.79 Violations (ROF) = 3 TPSA = 249.20 Å²
Isorhamnetin-3-o-galactoside	−11.923	−57.46	MW (<500 Da) = 478.40 g/mol Number of HB acceptor (10) = 12 Number of HB donor (<5) = 7 Log *P*(<5) = 1.21 Violations (ROF) = 2 TPSA = 199.51 Å²
Quercetin-3-o-galactoside	−10.729	−47.41	MW (<500 Da) = 464.38 g/mol Number of HB acceptor (<10) = 12 Number of HB donor (<5) = 8 Log *P*(<5) = 1.45 Violations (ROF) = 2 TPSA = 210.51 Å²
Kaempferol-3-o-glucoside	−10.501	−51.24	MW (<500 Da) = 448.38 g/mol Number of HB acceptor (10) = 11 Number of HB donor (<5) = 7 Log *P*(<5) = 0.53 Violations (ROF) = 2 TPSA = 190.28 Å²
Abiraterone acetate (control ligand)	−7.052	−34.38	MW (<500 Da) = 391.55 g/mol Number of HB acceptor (<10) = 3 Number of HB donor (<5) = 0 Log *P*(<5) = 3.74 Violations (ROF) = 1 TPSA = 39.19 Å²

MW, molecular weight; ROF, rule of five; TPSA, topological PSA.

**Figure 3:**
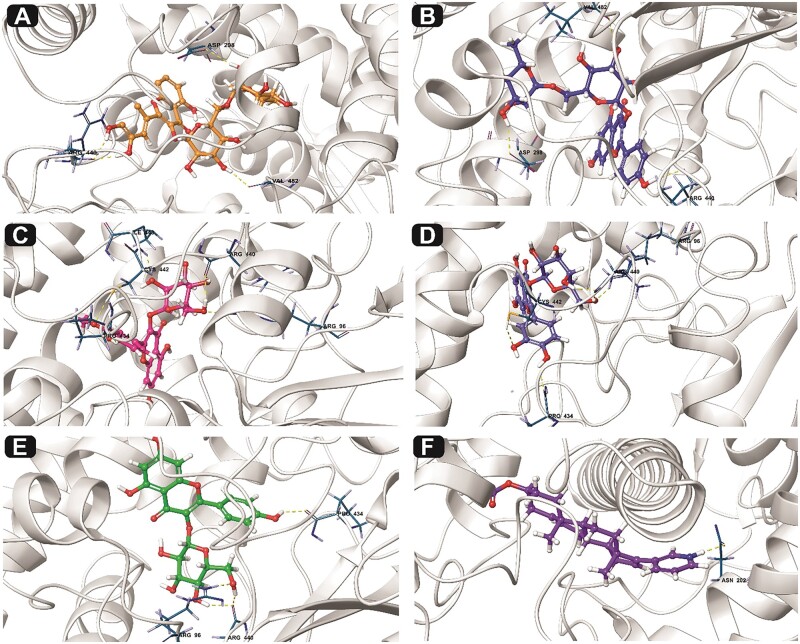
interaction profile of the CYP17A1 lyase–ligand complexes after molecular docking studies in 3D. Interactions are shown in dotted lines. (**a**) Quercetin-3-o-rutinoside-CYP_17_A_1_ lyase complex, (**b**) kaempferol-3-o-rutinoside—CYP_17_A_1_ lyase complex, (**c**) isorhamnetin-3-o-galactoside—CYP_17_A_1_ lyase complex, (**d**) quercetin-3-o-galactoside—CYP_17_A_1_ lyase complex, (**e**) kaempferol-3-o-glucoside—CYP_17_A_1_ lyase complex, and (**f**) AA-CYP_17_A_1_ lyase complex.

Based on the docking output, the essential amino acids at the active site of CYP17A1Lyase that play a crucial role in intra and intermolecular bonding interactions, including hydrophobic, pi–pi stacking, and hydrogen bonding interactions, are PRO 434, CYS 442, ARG 440, ARG 96, VAL 482, ASP 298, ILE 443, and ASN 202 as shown in Fig. 3. Quercetin-3-o-rutinoside, commonly known as Rutin, shows the highest binding energy of −14.361 Kcal/mol and exhibited non-covalent binding interactions with the following amino residues: ARG 440, ASP 298, and VAL 482 at the catalytic site of CYP_17_A_1_ lyase. The anti-cancer property of Rutin against different cancer cell lines has been well documented [44]. This result is consistent with an *invivo* study focused on the anti-proliferative potential of Rutin against cervical cancer cell, where Rutin significantly decrease cell viability in HPV-C33A cervical cancer cells through cell arrest and apoptosis induction [45]. Kaempferol-3-o-rutinoside had a binding score of −12.645 Kcal/mol while simultaneously establishing HBs with ASP 298, ARG 440, and VAL 482 in the defined active site of CYP17A1Lyase.

Furthermore, following the docking analysis, isorhamnetin-3-o-galactoside and quercetin-3-o-galactoside had a good binding orientation with binding affinities of −11.923 and −10.729 Kcal/mol, respectively. The two ligands interact with five amino acids: PRO 434, ILE 443, CYS 442, ARG 440, and ARG 96 at the target protein’s active site (CYP_17_A_1_ lyase). Additionally, kaempferol-3-o-glucoside establishes hydrophobic and hydrogen bonding interactions with PRO 434, ARG 96, and ARG 440. The reference ligand, AA, is an FDA drug used to treat metastatic CRPC cells. AA’s mechanism of action is based on the inhibition of CYP_17_A_1_ lyase, making it the most suitable reference compound. AA had the lowest binding energy of −7.052 Kcal/mol. AA bind with the heme component of cytochrome P450, thereby inhibiting its catalytic activity. This may also be the probable mechanism of compounds from Almond *P. dulcis.* Thus, the docking output predicted that the screened compounds exhibited relatively higher binding energy than the reference ligand.

**Figure 4:**
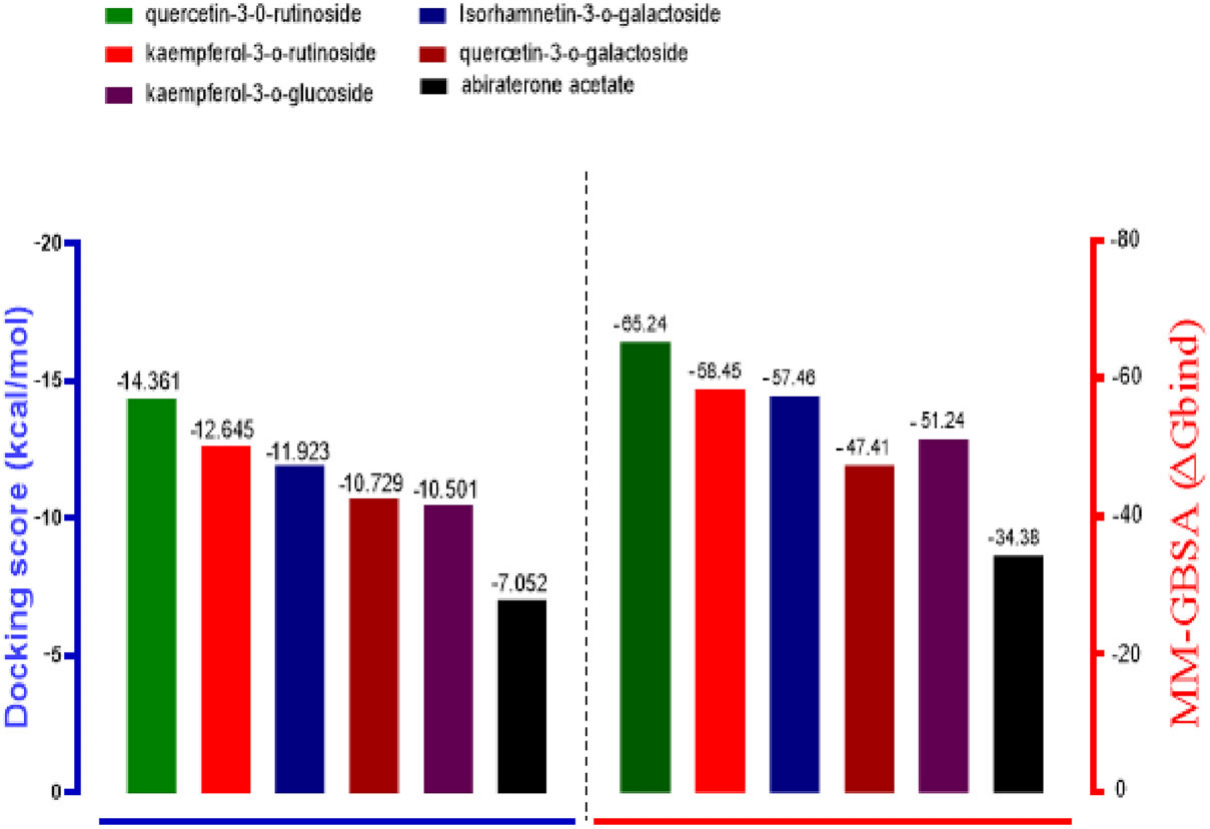
graphical representation of the molecular docking score and prime/MM-GBSA binding energy (ΔGbind) of *P. dulcis* compounds and the reference ligand. The left frame (blue) signifies the docking score, while the right edge (red) shows the MM-GBSA binding energy.

Molecular mechanics generalized born surface area (MM-GBSA) is a computational thermodynamics method used to estimate the binding affinity of compounds. Previous studies have reported that MM-GBSA incorporated in the Prime module of the Schrodinger suite provides a reliable statistical post-docking analysis of docked complexes and the more negative score corresponds to the higher binding. The relative free binding energy for quercetin-3-o-rutinoside, kaempferol-3-o-rutinoside, isorhamnetin-3-o-galactoside, quercetin-3-o-galactoside, kaempferol-3-o-glucoside and AA are −65.24, −58.45, −57.46, −47.41, −51.24, and −34.38, respectively, as shown in Fig. 4. The MM-GBSA result further confirms the higher binding energy of the selected bioactive compounds compared with the reference compound.

### Evaluation of ADMET properties

The *in silico* ADMET test was carried out to determine the pharmacokinetics and toxicological profile of the compounds [46]. The results of the ADMET profiling of all the hit compounds and the standard have been represented in Table 2.

**Table 2:** pharmacokinetic predictions for *P. dulcis* compounds and the reference ligand

Models	Quercetin-3-o-rutinoside	Kaempferol-3-o-rutinoside	Isorhmanetin-3-o-galactoside	Quercetin-3-o-galactoside	Kaempferol-3-o-glucoside	Abiraterone acetate
Ames mutagenesis	+	+	−	+	+	−
Acute oral toxicity (c)	II	III	III	III	III	III
BBB	−	−	−	−	−	+
Biodegradation	−	−	−	−	−	−
Caco-2 permeability	−	−	−	−	−	+
Carcinogenicity	−	−	−	−	−	−
CYP1A2 inhibition	+	−	−	−	−	−
CYP2C19 inhibition	−	−	−	−	−	+
CYP2C9 inhibition	−	−	−	−	−	−
CYP2C9 substrate	−	−	−	−	−	−
CYP2D6 inhibition	−	−	−	−	−	−
CYP2D6 substrate	−	−	−	−	−	−
CYP3A4 inhibition	+	−	−	−	−	+
CYP3A4 substrate	−	+	+	+	+	+
CYP inhibitory promiscuity	+	−	−	−	−	+
Hepatotoxicity	+	+	+	+	+	−
Human either-a-go-go inhibition	−	−	−	−	−	+
Human intestinal absorption	+	+	+	+	+	+
Human oral bioavailability	−	−	−	−	−	−
Acute oral toxicity	2.375795	2.350707	3.105423	3.075513	2.818906	2.695413
*P*-glycoprotein inhibitor	−	−	−	−	−	+
*P*-glycoprotein substrate	−	+	−	−	−	−
Plasma protein binding	1.161574	0.819224	0.829031	0.899552	0.823478	0.918855
Subcellular localization	Mitochondria	Mitochondria	Mitochondria	Mitochondria	Mitochondria	Mitochondria
UGT catalyzed	+	−	+	+	−	−
Water solubility	−2.99937	−2.7724	−2.31765	−2.44888	−2.44888	−5.25104

To determine the absorption of the drug candidates, human intestinal absorption and P-glycoprotein inhibition of the compounds were determined. Interestingly, all the ligands were found to possess positive value for human intestinal absorption. None except the standard was an inhibitor of P-glycoprotein, which is known to facilitate the transport of drugs in the cell. These results indicate excellent absorption for all five chosen compounds and are promising better than the standard.

In the distribution part, two pharmacological parameters—the blood–brain barrier (BBB) and p-glycoprotein substrate were put into consideration to determine whether the compounds could permeate the BBB and could act as a substrate of p-glycoprotein [47]. Since the value of the BBB is negative, this indicates that the drug candidates could not pass through the BBB, and hence the central nervous system is protected from their action. Furthermore, all compounds, except for kaempferol-3-o-rutinoside, are non-substrate of p-glycoprotein. Accordingly, kaempferol-3-o-rutinoside distribution may be limited by the mediation of p-glycoprotein efflux.

The superfamily of Cytp450 carries out the metabolism of drugs. The inhibition of these enzymes is known to affect the metabolism and excretion of the drug candidates and could result in bio-accumulation [48]. In the ADMET table shown in Table 2, the only compound with inhibition of the Cytp_450_ superfamily (Cytp_1_A_4_ and Cytp_3_A_4_) is quercetin-3-o-rutinoside. However, considering the standard drug AA also posed an inhibitor of two Cytp_450_ families (Cytp_2_C_19_ and Cytp_3_A_4_), the negative influence of quercetin-3-o-rutinoside on the metabolic activity may be minimal.

The safety of drugs for use is mainly dependent on their pharmacodynamic profile. Most drugs fail to pass through clinical trials because of unacceptable toxicological properties. Therefore, Ames mutagenicity, hepatotoxicity, human ether-a-go-go inhibition, and carcinogenicity of the compounds were examined. Disappointingly, all the compounds prove to be hepatotoxic and also exhibiting Ames mutagenicity. However, the hepatotoxic effect of the compounds may be reduced with the optimal dosage of the drugs. Furthermore, the functional group of the compounds could be modified during drug development process to yield drug candidates with an acceptable biosafety profile. Nonetheless, it is encouraging that all the five compounds were non-carcinogenic.

### MD simulation of CYP_17_A_1_ lyase–quercetin-3-o-rutinoside complex

**Figure 5:**
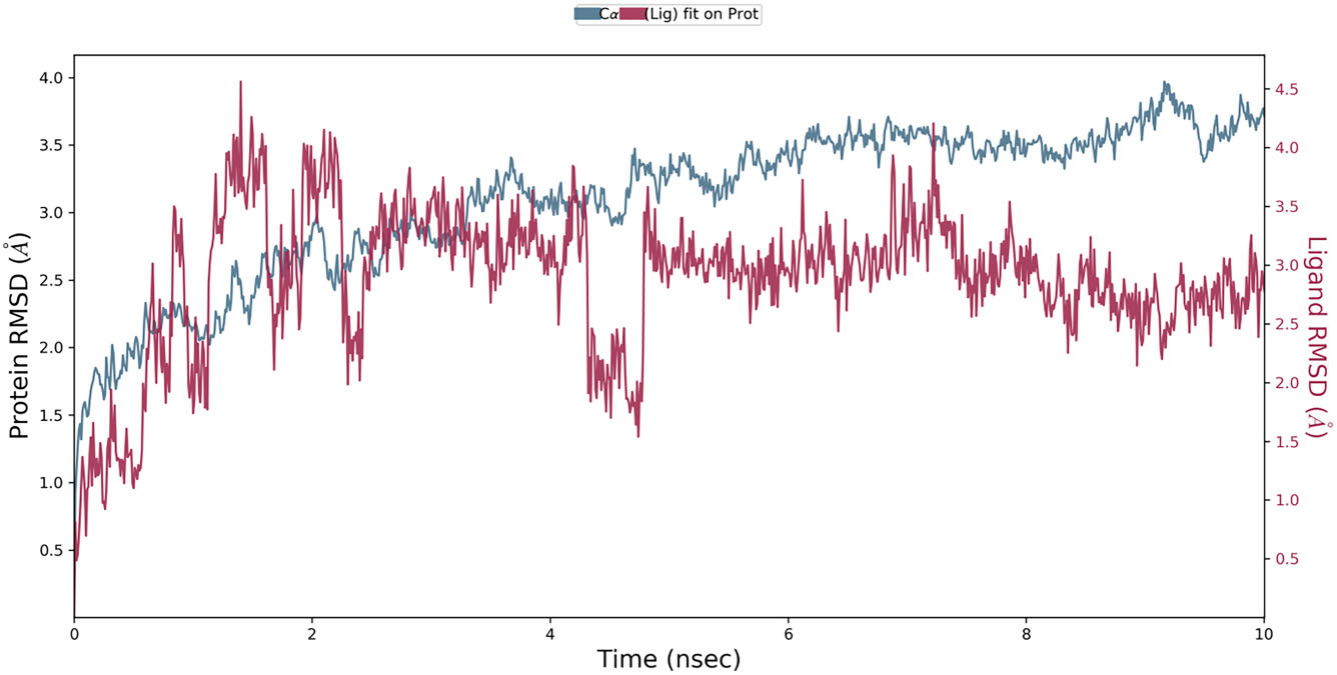
the RMSD of the protein receptor and ligand.

**Figure 6:**
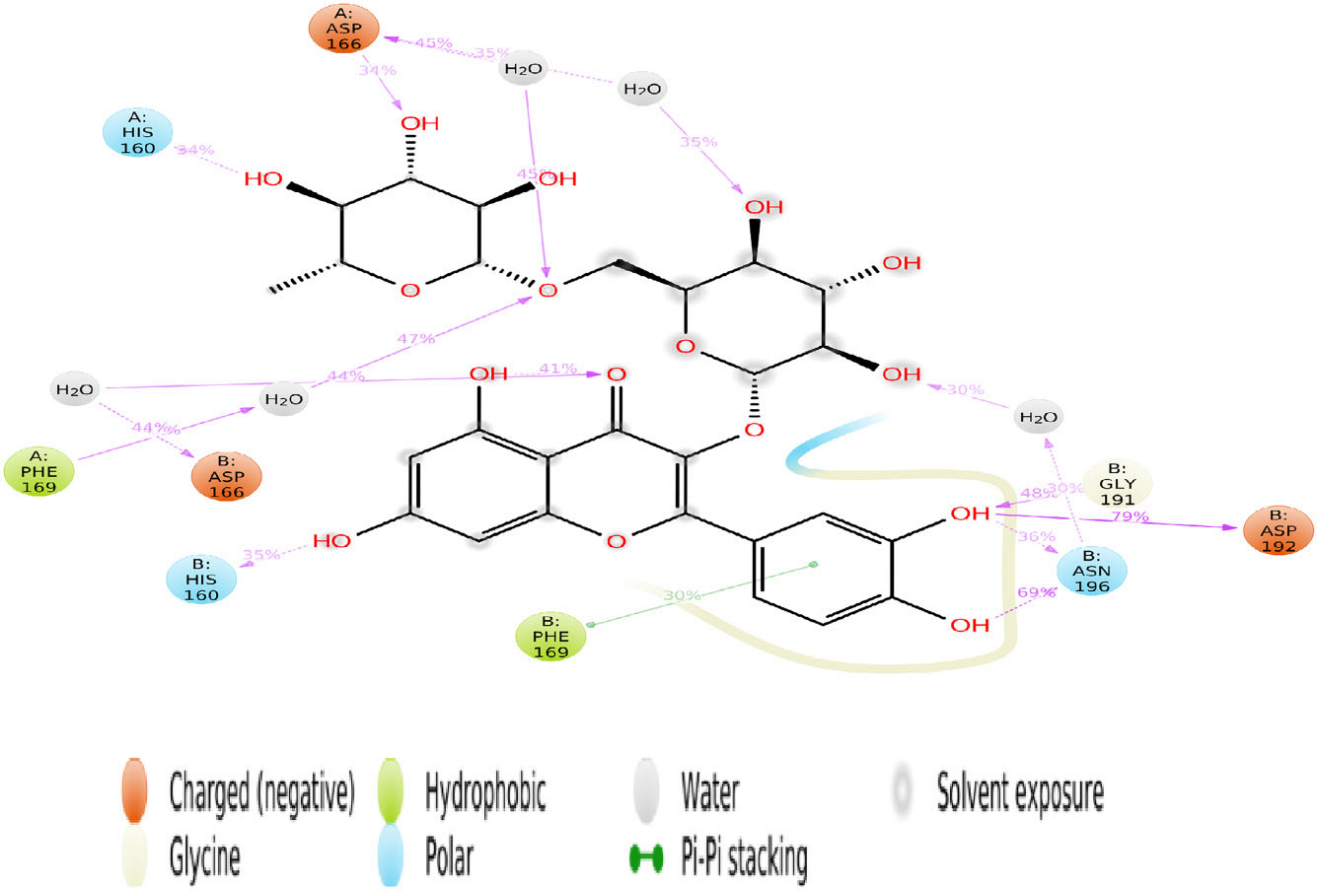
schematic of detailed ligand atom interactions with the protein residues.

The average change in the atomic displacement of the superimposed complex concerning the reference frame was measured considering their RSMD. The boundaries of the trajectory were calculated. The generated RMSD plot indicates equilibrium or stability between the superimposed complex, which depends on the binding interaction and energy between the protein receptor and ligand. At a simulation time of 3 ns, there was conformational stability between the complexes at a crystallography resolution of 3.0–3.2 Å. This change is acceptable for a small, globular protein. This also signifies the protein conformational change and linear relationship at the binding of the ligand during the simulation (Fig. 5). We mapped out the HB interaction between the atomic ligand and the amino residues of the protein receptor. It represented the interaction that occurred more than 30% of the simulation time in the trajectory from 0.0 to 10 ns. The interaction amino residues were ASP 166, HIS 160, PHE 169, HIS 160, ASP 166, ASN 196, ASP 192, and GLY 191, respectively (Fig. 6). The hydrophobic atoms were PHE 169 from both A and B chains, respectively. An intriguing observation was the interaction between water and the PHE 169 despite its hydrophobicity.

**Figure 7:**
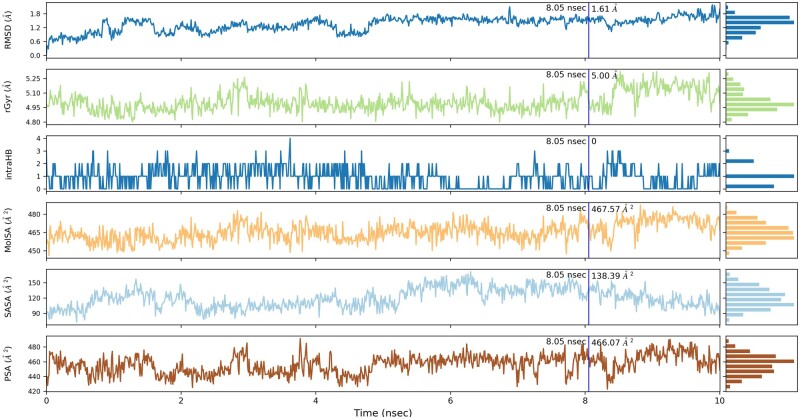
the ligand properties of the interaction.

Individual ligand crystallographic properties characterizing the interaction were summarized. At the time simulation of 8.05 ns, the ligand RSMD was 1.61 Å and the radius of gyration was 5.0 Å from the baseline trajectory. No internal HBs of the ligand were detected in the interaction. The molecular surface area (MolSA), whose value is equivalent to van der Waals surface area, was 467.57 A^2^ and solvent accessible surface area (SASA) contributed by water molecule was 138.39 A^2^. Finally, the polar surface area (PSA) contributed only by oxygen and nitrogen atoms was 466.07 A^2^ (Fig. 7).

### Ligand torsion profile

**Figure 8:**
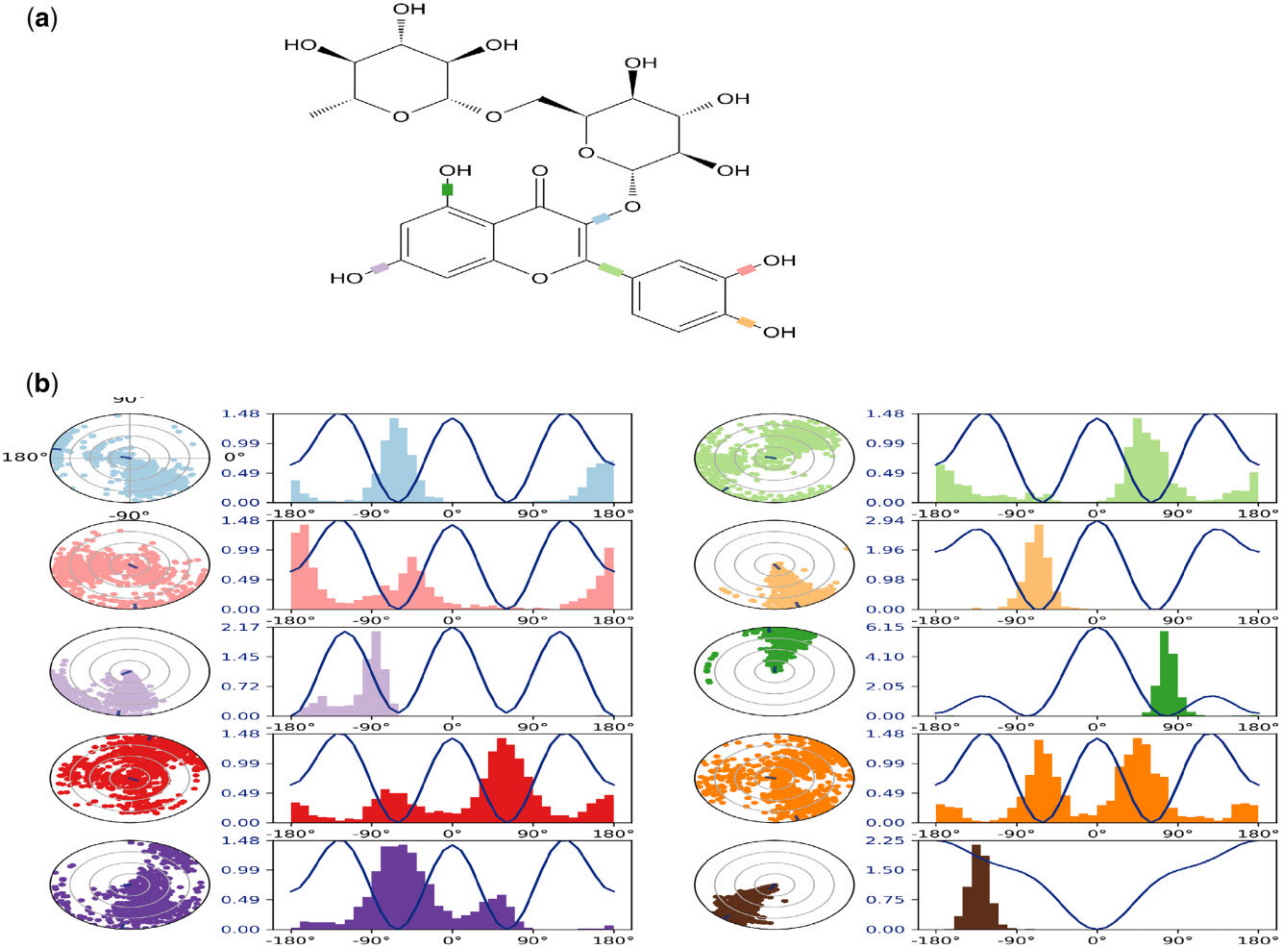
ligand torsion potential. (**a**) Representation of the rotating ligand during the simulation process. (**b**) The obtained radial plots showing the torsion potential of the ligand. The potential values are on the left *Y*-axis of the chart and are expressed in kcal/mol.

**Figure 9:**
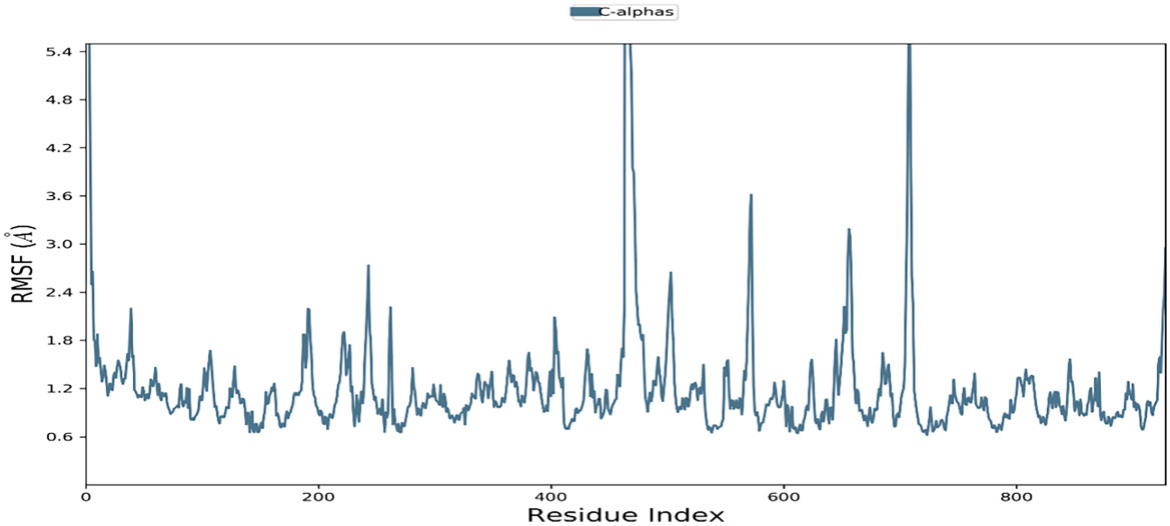
protein RSMF. The peak represents the RSMF of each amino residue.

The conformational evolution of the rotatable bond in the ligand throughout the simulation trajectory, the 2D schematic of a ligand with color-coded rotatable bonds was first obtained (Fig. 8a), before the generated rotatable bond, a dial (radial) plot accompanies torsion and bar plots of the same color. The radial plots describe the conformation of the torsion throughout the simulation. The center of the radial plot represents the start of the simulation and the time evolution is plotted outwards. The provided bar plots summarize the data on the dial plots by showing the probability density of the torsion. The ligand showed torsional potential during the round of simulation. The chart offered the conformational strain the ligand undergoes to maintain a protein-bound conformation (Fig. 8b). We examined the regions in the protein chain that fluctuates most during the simulation time. This was calculated considering their root mean square fluctuation (RSMF) as local changes were characterized. Mostly, the unstructured loop regions of the protein had more fluctuations (high peak) than the structured and rigid alpha helices and beta strands (Fig. 9).

**Figure 10:**
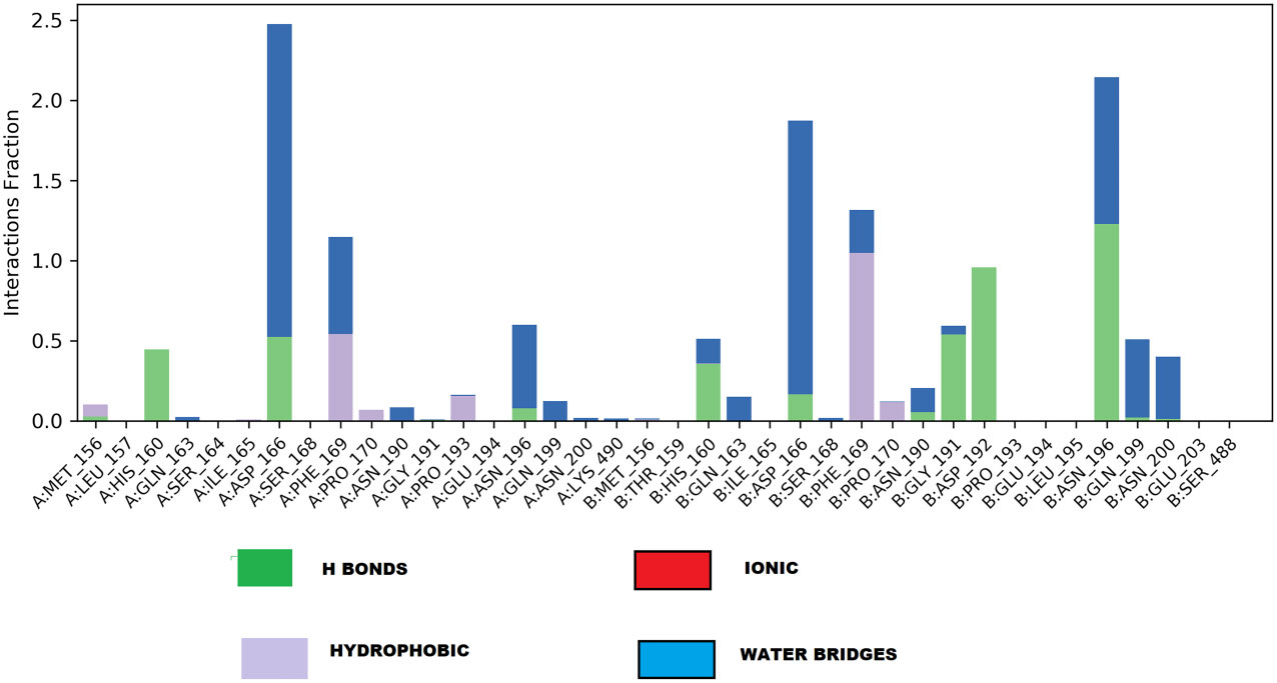
protein–ligand interactions (or “contacts”) of the amino acids. The interaction is categorized into four types: HBs, hydrophobic, ionic, and water bridges.

**Figure 11:**
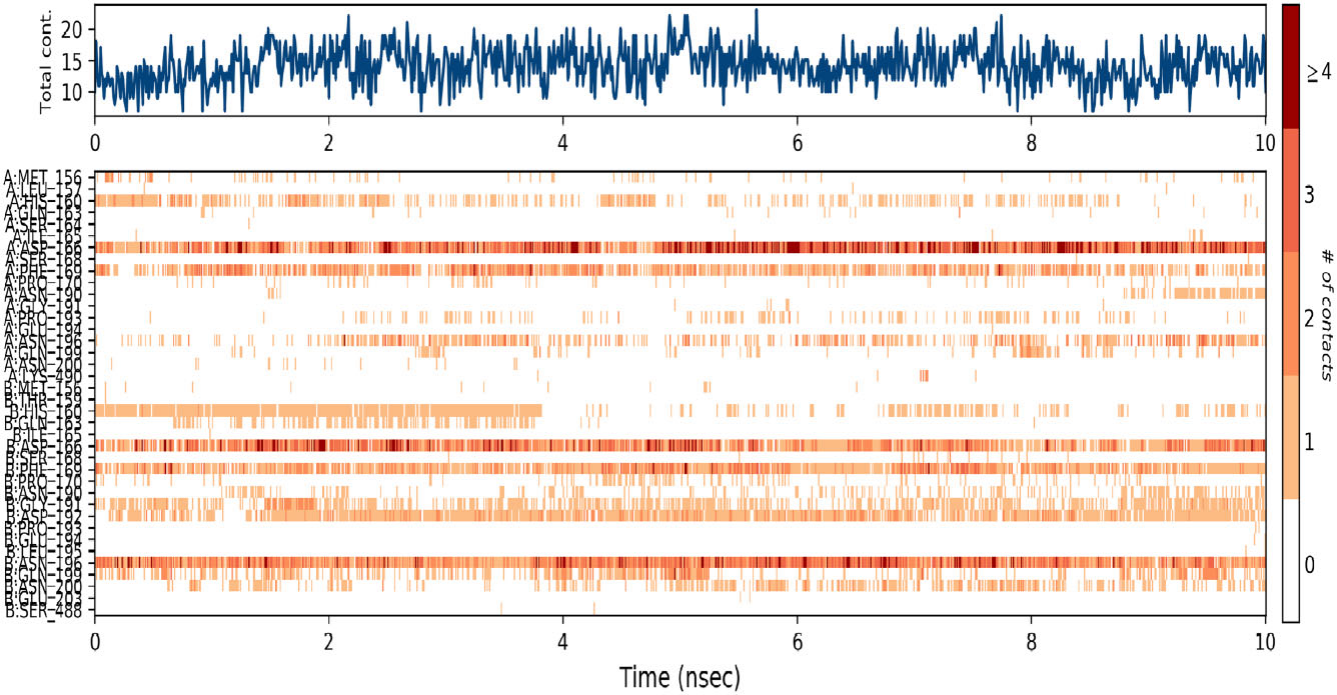
the top panel shows the number of specific contacts the protein makes with the ligand throughout the trajectory. The bottom panel shows which residues interact with the ligand in each trajectory frame. Some residues make more than one specific contact with the ligand, which is represented by a darker shade of orange, according to the scale to the right of the plot.

It is imperative to represent the type of interactions that each amino residue of the protein exhibit with the ligand throughout the simulation (Fig. 10). Considering the types of interactions and contacts exhibited by each deposition, a heat map was generated, summarizing their H-bonds, hydrophobic, ionic, and water bridges (Fig. 11). The values of the bar charts are normalized throughout the trajectory. Most residues make multiple contacts of the same subtype with the ligand. The molecular docking and dynamic simulation results obtained in this present study was observed to be consistent with the findings of Gurung et al. [49] who reported the molecular docking and dynamics simulation of β-bourbonene from *Ficus carica* as inhibitor of PCa targets.

## Conclusion

The bioactive compounds from Almond *P.dulcis* demonstrated higher selective inhibitory potential against CYP_17_A_1_lyase activity and favorable pharmacological properties when compared with AA. This therapeutic profile of the compounds could lower the risk for undesirable side effects, particularly control of corticosteroid production in humans compared with AA. Thus, proposing Almond *P. Dulcis* compounds as new promising molecules to develop an improved drug against PCa. However, there is a need for further studies such as *invivo* and *invitro* to validate the efficacies of the Almond *P. dulcis* bioactive compounds in CRPC therapy.

## Author contributions

D.A.O. and T.A.B.: conceptualization, software, and formal analysis. T.A.B. and D.S.B.: methodology. O.A.S.: validation. D.S.B. and A.A.: data curation. T.A.B., A.A., and O.A.S.: writing—original draft preparation. T.A.B., A.A., D.A.O., G.E.B., and D.S.B.: writing—review and editing. D.A.O. and G.E.B.: supervision. All authors read and approved the final manuscript.

## Funding

This research received no external funding.

*Conflict of interest statement*. No potential conflict of interest was reported by the authors.

## Data Availability

The data underlying this article are available in the article and in its online supplementary material
